# Dual‐specificity phosphatase 6 plays a critical role in the maintenance of a cancer stem‐like cell phenotype in human endometrial cancer

**DOI:** 10.1002/ijc.32965

**Published:** 2020-03-26

**Authors:** Masaya Kato, Ichiro Onoyama, Sachiko Yoshida, Lin Cui, Keiko Kawamura, Keisuke Kodama, Emiko Hori, Yumiko Matsumura, Hiroshi Yagi, Kazuo Asanoma, Hideaki Yahata, Atsuo Itakura, Satoru Takeda, Kiyoko Kato

**Affiliations:** ^1^ Department of Obstetrics and Gynecology School of Medical Sciences, Kyushu University Fukuoka Japan; ^2^ Department of Obstetrics and Gynecology School of Medical Sciences, Juntendo University Tokyo Japan

**Keywords:** DUSP6, cancer stem‐like cell, endometrial cancer, ERK1/2, Akt

## Abstract

The prognosis of patients with high‐grade or advanced‐stage endometrial cancer remains poor. As cancer stem‐like cells (CSCs) are thought to be associated with endometrial cancers, it is essential to investigate the molecular mechanisms that regulate endometrial CSCs. Dual‐specificity phosphatase 6 (DUSP6) functions as a negative‐feedback regulator of MAPK–ERK1/2 signaling, but its role in endometrial cancer remains unknown. We investigated whether DUSP6 is involved in cancer cell stemness using endometrial cancer cell lines and specimens from endometrial cancer patients. DUSP6 induced the expression of CSC‐related genes including ALDH1, Nanog, SOX2 and Oct4A, increased the population of cells in the G0/G1 phase, and promoted sphere formation ability. DUSP6 knockdown resulted in reduced cell invasion and metastasis, whereas DUSP6 overexpression inhibited apoptosis under serum‐free conditions. Moreover, DUSP6 decreased phosphorylated ERK1/2 and increased phosphorylated Akt levels, which potentially induces CSC features. In patients with endometrial cancers, DUSP6 expression was determined using immunohistochemistry, and based on the results, the patients were dichotomized into high‐ and low‐DUSP6‐expression groups. Progression‐free survival and overall survival were significantly shorter in the high‐DUSP6‐expression group. These results suggest that DUSP6 has potential value as a biomarker of CSCs and as a target of therapies designed to eliminate CSCs in endometrial cancer.

## Abstract 2

**What's new?**

Although cancer stem‐like cells (CSCs) are involved in human endometrial cancers, the underlying molecular mechanisms and biomarkers for CSCs in endometrial cancers remain elusive. Here, the authors found that DUSP6 plays an important role in regulating endometrial CSC phenotypes by increasing self‐renewal ability and starvation resistance. DUSP6 expression was required for inducing invasion and metastasis and resulted in ERK1/2 dephosphorylation and Akt phosphorylation, which potentially contribute to the promotion of CSC phenotypes. As DUSP6 expression was also positively associated with worse progression‐free and overall survival, DUSP6 represents a potential biomarker for endometrial CSCs and a therapeutic target in endometrial cancers.

**Abbreviations**
BrdU: bromodeoxyuridine
CSC: cancer stem‐like cell
DUSP6: dual‐specificity phosphatase 6
ELDA: extreme limiting dilution analysis
EMT: epithelial–mesenchymal transition
ERK: extracellular signal‐regulated kinase
MAPK: mitogen‐activated protein kinase
SP: side‐population
TIF: tumor‐initiating cell frequency

## Introduction

Endometrial cancer is the seventh most common cancer in women. Approximately 382,100 new cases per year are diagnosed worldwide, and 89,900 patients per year die from the disease.^1^, ^2^ Whereas patients with low‐grade and early‐stage endometrial cancer have good prognoses, the outcomes of those with high‐grade and metastatic or recurrent cancer remain poor despite the advances in diagnostic and therapeutic interventions. Although chemotherapy regimens involving platinum and taxanes are used to treat advanced cases, their effects are limited.^3^ To eliminate cancer in patients with poor prognoses, further investigation of the tumorigenic mechanisms is needed.

Cancer stem‐like cells (CSCs), a subset of tumor cells that can reconstitute cancer tissues, are responsible for cancer progression, metastasis and resistance to therapies.^4^, ^5^, ^6^, ^7^ CSCs have been identified and validated in various hematological cancers and solid tumors.^8^, ^9^, ^10^, ^11^ Although CSC involvement has been demonstrated in human endometrial cancers as well,^12^ effective biomarkers and the molecular mechanisms of CSCs in endometrial cancers remain unknown. We also previously demonstrated that endometrial cancer side‐population (SP) cells, which are characterized by their ability to efflux Hoechst 33342 dye, exhibit CSC features,^13^ including the potential to differentiate into mesenchymal cells and an association with epithelial–mesenchymal transition (EMT).^14^, ^15^ Although dual‐specificity phosphatase 6 (DUSP6), a member of the dual‐specificity phosphatase family, was reported to prevent breast CSC formation, it was also found to be induced during EMT^16^ and to be associated with an invasive phenotype, metastasis and recurrence in various malignant cancers.^17^, ^18^, ^19^, ^20^, ^21^, ^22^, ^23^ DUSP6 selectively inactivates ERK1/2 *via* dephosphorylation of both phosphothreonines and phosphotyrosines, functioning as a negative‐feedback regulator of MAPK–ERK1/2 signaling.^24^, ^25^, ^26^ However, the role of DUSP6 in endometrial CSCs is unknown.

In our study, we demonstrated that DUSP6 supports a CSC phenotype in endometrial cancer cells. We showed that DUSP6 increases phosphorylated Akt and decreases phosphorylated ERK1/2 levels, thus increasing CSC‐related gene expression and the proportion of cells in the G0/G1 phase, as well as inducing a malignant phenotype. We found that DUSP6 was associated with short progression‐free survival (PFS) and overall survival (OS) in patients with endometrial cancer and thus may be a therapeutic target in the CSCs of patients with endometrial cancer.

## Materials and Methods

### Cell culture

The human endometrial cancer cell lines Hec1 (RRID: CVCL_1274) and HHUA (RRID: CVCL_3866) were purchased from JCRB Cell Bank and RIKEN Bioresource Center, respectively. All cells were routinely verified to be mycoplasma free and were authenticated by short tandem repeat profiling within the last 3 years. Cells were maintained in Dulbecco's modified Eagle's medium (DMEM; Nacalai Tesque, Kyoto, Japan) supplemented with 10% fetal bovine serum (FBS) and 1% penicillin/streptomycin (Gibco, New York, NY) at 37°C in a humidified incubator containing 5% CO_2_.

### Plasmids

The lentiviral expression vectors used for DUSP6 overexpression have been described previously.^27^ The coding region of the human DUSP6 gene was amplified by PCR, verified by DNA sequencing and then inserted into the lentiviral expression vectors. The empty vectors were used as controls. The lentiviral miR‐E‐based expression vector pLKO1 was a gift from the laboratory of Michael R. Green. Two short hairpin RNAs (shRNAs) directed against DUSP6 were used in all experiments, and their sequences are sh‐DUSP6_1: CTTGGACGTGTTGGAGGAATT (TRCN0000002437) and sh‐DUSP6_2: CAGTAAGTTCCAAGCCGAGTT (TRCN0000002438). The empty vectors were used as controls.

### Transfection and packaging of lentiviruses

Lentiviruses were packaged using HEK293T cells. HEK293T cells were transfected with the lentiviral vectors using Lipofectamine 2000 (Thermo Fisher Scientific), as described previously.^27^ At 48 hr post‐transfection, the culture supernatant containing the lentiviruses were collected and passed through 0.45 μm filters. The lentivirus‐containing supernatant was added to cells in the presence of polybrene at a final concentration of 8 ng/μl.

### Protein extraction and Western blotting

The cells were washed in ice‐cold PBS and lysed using RIPA buffer supplemented with a protease inhibitor cocktail (Sigma‐Aldrich, St. Louis, MO) and phosphatase inhibitor cocktail (Nacalai Tesque). For Western blot analysis, 10 μg total protein were loaded and run on 7.5–15% precast gradient polyacrylamide gels. The gels were transferred to nitrocellulose membranes at 20 V overnight. Membranes were blocked in 5% milk or Blocking One‐P (Nacalai Tesque) for 20 min and incubated with the primary antibody at 4°C overnight. The primary antibodies used for Western blot analysis are listed in Supporting Information Table S1. After washing, the membranes were incubated for 1 hr at room temperature with the secondary antibody. Specific protein bands were detected using the SuperSignal West Pico/Dura Chemiluminescent Substrate (Thermo Fisher Scientific, Waltham, MA).

### ALDEFLUOR assay

Hec1 cells were harvested using 0.25% trypsin/1 mM EDTA (Nacalai Tesque) solution and evaluated for aldehyde dehydrogenase (ALDH) activity using the ALDEFLUOR assay kit per the manufacturer's instructions (STEMCELL Technologies, Vancouver, Canada). Flow cytometry was conducted using the FACS Caliber or FACSVerse (BD Biosciences, San Jose, CA).

### Bromodeoxyuridine‐labeling assay

Cells were seeded on 10 cm dishes at 1 × 10^5^/ml and grown overnight. The cells were incubated with 10 μM bromodeoxyuridine (BrdU) for 45 min, after which they were washed with PBS and trypsinized. The cells were fixed and incubated with an anti‐BrdU antibody and 7‐AAD staining solution provided in the BrdU Flow Kit (BD Pharmingen, Franklin Lakes, NJ), according to the manufacturer's instructions. Flow cytometry was conducted using the FACSVerse (BD Biosciences).

### Sphere formation assay

Cells were trypsinized and resuspended in serum‐free DMEM/F12 (Nacalai Tesque) supplemented with B‐27, 20 ng/ml EGF and 10 ng/ml bFGF, and 1–3 × 10^3^ cells/well were seeded into 24‐well ultra‐low adhesion plates (Corning, Corning, NY). The cells were cultured for 8 days, and then spheres were counted under the BZ‐X710 microscope (Keyence, Osaka, Japan). The spheres were dissociated using accutase (Sigma‐Aldrich) at 37°C for 20 min and regularly triturated by pipetting to generate single cells and replated into new low‐attachment plates.

### Animals

All animal procedures were conducted under the guidelines approved by the Animal Care and Use Committee of Kyushu University (approval reference A30‐131‐1). The study was conducted according to the Animal Research Reporting *In Vivo* Experiments (ARRIVE) guidelines.

### Limiting dilution assay *in vivo*

Six‐week‐old female NOD/SCID mice (CLEA Japan) were used as hosts for the *in vivo* limiting dilution assay. In the limiting dilution assay, 5 × 10^4^, 2 × 10^4^, 1 × 10^4^ and 5 × 10^3^ cells were injected into the subcutaneous tissue of both shoulders and buttocks, respectively. Mice were monitored weekly for tumor formation or any sign of illness/weakness and were sacrificed 8 weeks later. The tumor‐initiating cell frequency (TIF) was calculated using extreme limiting dilution analysis (ELDA) software (http://bioinf.wehi.edu.au/software/elda/).

### Cell migration and invasion assay

Cells invasion and migration assays were performed using cell culture inserts with an 8.0 μm pore size with or without extracellular matrix. A total of 5.0 × 10^4^ cells were suspended in 100 μl serum‐free DMEM and added to the upper insert, and the lower well was filled with 750 μl DMEM containing 10% FBS. After 36 hr of incubation, residual cells were wiped with cotton‐tipped swabs. Invasive cells were fixed with methyl alcohol for 2 min and stained with Diff‐Quick (Sysmex, Kobe, Japan). The invaded cells were observed under the BZ‐X710 microscope (Keyence).

### *In vivo* lung metastasis model

Cells (1 × 10^6^) stably expressing luciferase were injected into the tail vein (*n* = 8 per group) of female BALB/c‐*nu/nu* nude mice (CLEA Japan, Tokyo, Japan). Tumor growth was evaluated by measuring luciferase activity (bioluminescence intensity) in the whole body in mice 35 days after cell injection. d‐Luciferin (100 mg/kg) was injected subcutaneously into mice under isoflurane anesthesia, and 15 min later, luciferase activity was evaluated using the IVIS imaging system (Perkin Elmer, Waltham, MA). Mice were euthanized 35 days after cell injection, and their lungs were extracted. The amount of lung metastasis was quantitated using the IVIS imaging system and confirmed by hematoxylin and eosin staining.

### Liver metastasis model *in vivo*

Cells (1 × 10^6^) stably expressing luciferase were implanted into the subcapsular region of the spleen (*n* = 7 each group) of female BALB/c‐*nu/nu* nude mice (CLEA Japan). The tumor volumes were evaluated by measuring luciferase activity (bioluminescence intensity) in the whole body of mice once a week using the IVIS imaging system. Mice were euthanized 14 days after cell injection, and livers were extracted to quantitate the volumes of the liver metastases using the IVIS imaging system.

### Annexin V apoptosis assay

Cells were cultured in serum‐free DMEM. Apoptotic cell death was evaluated by annexin V and PI staining using the MEBCYTO Apoptosis Kit (MBL) according to the manufacturer's instructions. Fluorescent cells were analyzed using FACSVerse (BD Biosciences).

### Endometrial cancer tissues

Endometrial cancer specimens from 90 female patients who were treated at Kyushu University Hospital between 2004 and 2013 were evaluated, and patients with uterine cancers other than endometrial cancer were excluded. The median age of the patients was 58 years. All patients had undergone surgical resection without preoperative chemotherapy or radiation therapy. The cases were staged using the 2008 International Federation of Gynecology and Obstetrics staging system. Tumors were histologically classified as well‐differentiated (Grade 1), moderately differentiated (Grade 2) or poorly differentiated (Grade 3). Follow‐up information was obtained by reviewing the medical records. Informed consent was obtained from all patients prior to enrollment in the study. All patients involved in our study provided their signed informed consent. Our study was approved by the Ethical Committee of Kyushu University (approval number: 622‐00) and was conducted in accordance with the Declaration of Helsinki.

### Immunohistochemistry

Tissue sections (4 μm thick) used for immunohistochemistry (IHC) were obtained from formalin‐fixed, paraffin‐embedded blocks. The sections were deparaffinized and rehydrated. For antigen retrieval, the sections were incubated in antigen retrieval reagent, pH 9.0 (Nichirei Bioscience) and boiled for 10 min using a microwave. Endogenous peroxidases were blocked using 3% hydrogen peroxide in methyl alcohol for 15 min. The slides were incubated with a rabbit polyclonal DUSP6 antibody (Supporting Information Table S1) at a 1/50 dilution at 4°C overnight. The slides were washed and incubated with the secondary antibody at room temperature for 1 hr. The IHC signal was detected using DAB (Wako), and the nuclei were stained with hematoxylin. IHC staining was detected under the BZ‐X710 microscope (Keyence), and the total IHC score, calculated as the sum of the score for the proportion of positively stained cells and the score for staining intensity (Supporting Information Table S2), was used to evaluate each case. Cut‐off IHC scores were determined according to the median score, and the cases were divided into the low‐DUSP6‐expression (total score: 0–3 points) and high‐DUSP6‐expression (total score: 4–6 points) groups. At least two examiners evaluated all cases without prior knowledge of the clinicopathological data.

## Statistical analysis

To analyze the differences between two or more groups, Student's *t‐*test, Fisher's exact test, and ANOVA were used. When the ANOVA results were significant, the Tukey–Kramer method was used. Progression‐free survival (PFS) was defined as the time from surgery to the first radiological confirmation of progression, and overall survival (OS) as the time from surgery to death. PFS and OS curves were plotted using the Kaplan–Meier method and compared using the log‐rank test. All differences with a *p*‐value < 0.05 were considered statistically significant. Statistical analyses were performed using GraphPad Prism 7 (GraphPad Software, La Jolla, CA).

## Data availability

The data supporting the findings of our study are available from the corresponding author upon reasonable request.

## Results

### DUSP6 increases CSC‐related gene expression and contributes to maintaining the quiescent state and self‐renewal ability of endometrial cancer cells

**Figure 1:**
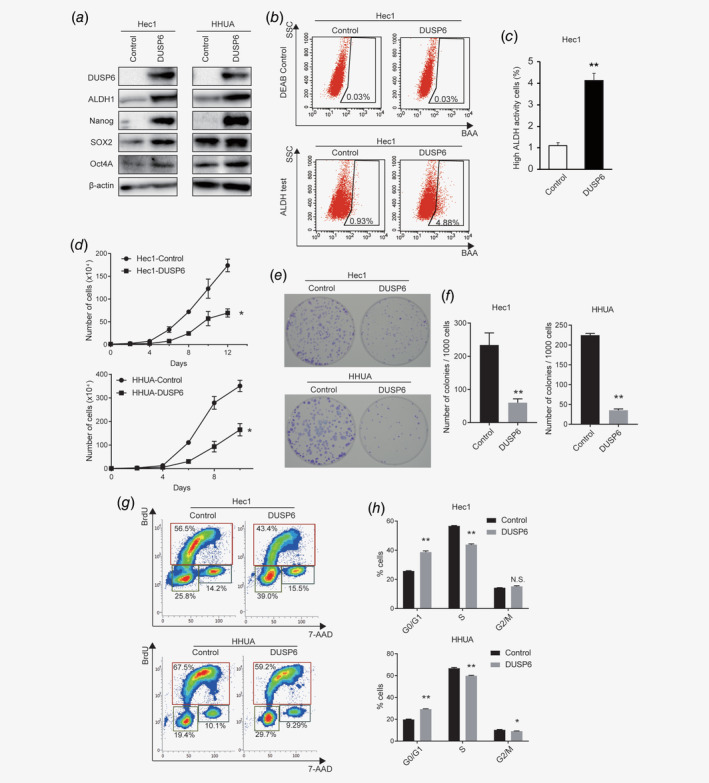
DUSP6 increases expression of CSC‐related genes and the number of cells in the G0/G1 phase. (*a*) Western blot analysis of pluripotency‐related and CSC‐related genes in Hec1 and HHUA cells. β‐Actin was also measured to ensure equal loading of a gel and a representative loading control for all sample is shown. (*b*) ALDEFLUOR assay to detect ALDH activity using flow cytometry in control and DUSP6‐overexpressing Hec1 cells. The numbers indicate the population of ALDH‐positive cells. (*c*) Bar graph showing the ALDH‐positive populations among control and DUSP6‐overexpressing Hec1 cells. (*d*) Growth curves of DUSP6‐overexpressing Hec1 and HHUA cells. A total of 1.0 × 10^4^ cells were plated in DMEM containing 10% FBS, and the number of cells was counted every other day. (*e*) Images of the colonies formed from DUSP6‐overexpressing Hec1 and HHUA cells. A total of 1.0 ×10^3^ single cells were cultured in a 6‐cm dish for 14 days. The resulting colonies were stained with Giemsa. (*f*) Bar graph showing the number of colonies that were ≥0.5 mm in diameter. (*g*) The proportions of control and DUSP6‐overexpressing Hec1 and HHUA cells in different cell‐cycle phases examined by flow cytometric BrdU‐labeling assay. The cells in the G0/G1, S and G2/M phases were gated. The numbers indicate the percentages of the cell population at each gate. (*h*) Bar graph quantifying the proportion of cells in each cell‐cycle phase. Data are representative of at least three independent experiments. Error bars indicate the standard deviation. *p < 0.05, **p < 0.01; N.S., not significant.

To analyze DUSP6 functions related to the stemness of tumor cells, human DUSP6 was overexpressed in the Hec1 and HHUA endometrial cancer cell lines, and the CSC population was examined. Overexpression of DUSP6 upregulated the expression of CSC‐related genes including Nanog, SOX2, Oct4A and ALDH1 (Fig. 1 *a*). In addition, we performed the ALDEFLUOR assay to measure ALDH activity, a marker of CSCs,^28^ and the results showed that ALDH activity was upregulated in DUSP6‐overexpressing cells (Figs. 1 *b* and 1 *c*). DUSP6 may stimulate the expression of CSC‐related genes in endometrial cancer cells.

We assessed the effect of DUSP6 on cell proliferation in medium containing 10% serum. Cell growth curves showed that the proliferation of DUSP6‐overexpressing cells was significantly decreased compared to control cells (Fig. 1 *d*). We also performed a focus assay. The colony size and the number of colonies ≥0.5 mm in diameter were significantly reduced in DUSP6‐overexpressing cells (Figs. 1 *e* and 1 *f*). Given that CSCs remain in the G0 phase of the cell cycle and remain quiescent for as long as needed,^29^ we investigated the effect of DUSP6 on the cell cycle. A BrdU‐labeling assay showed that the population of DUSP6‐overexpressing cells in the G0/G1 phase was increased, and that in the S phase was decreased (Figs. 1 *g* and 1 *h*). Thus, DUSP6 may increase the population of cancer cells in the quiescent state. Collectively, these results suggest that DUSP6 expression in endometrial cancer cells induces CSC characteristics.

**Figure 2:**
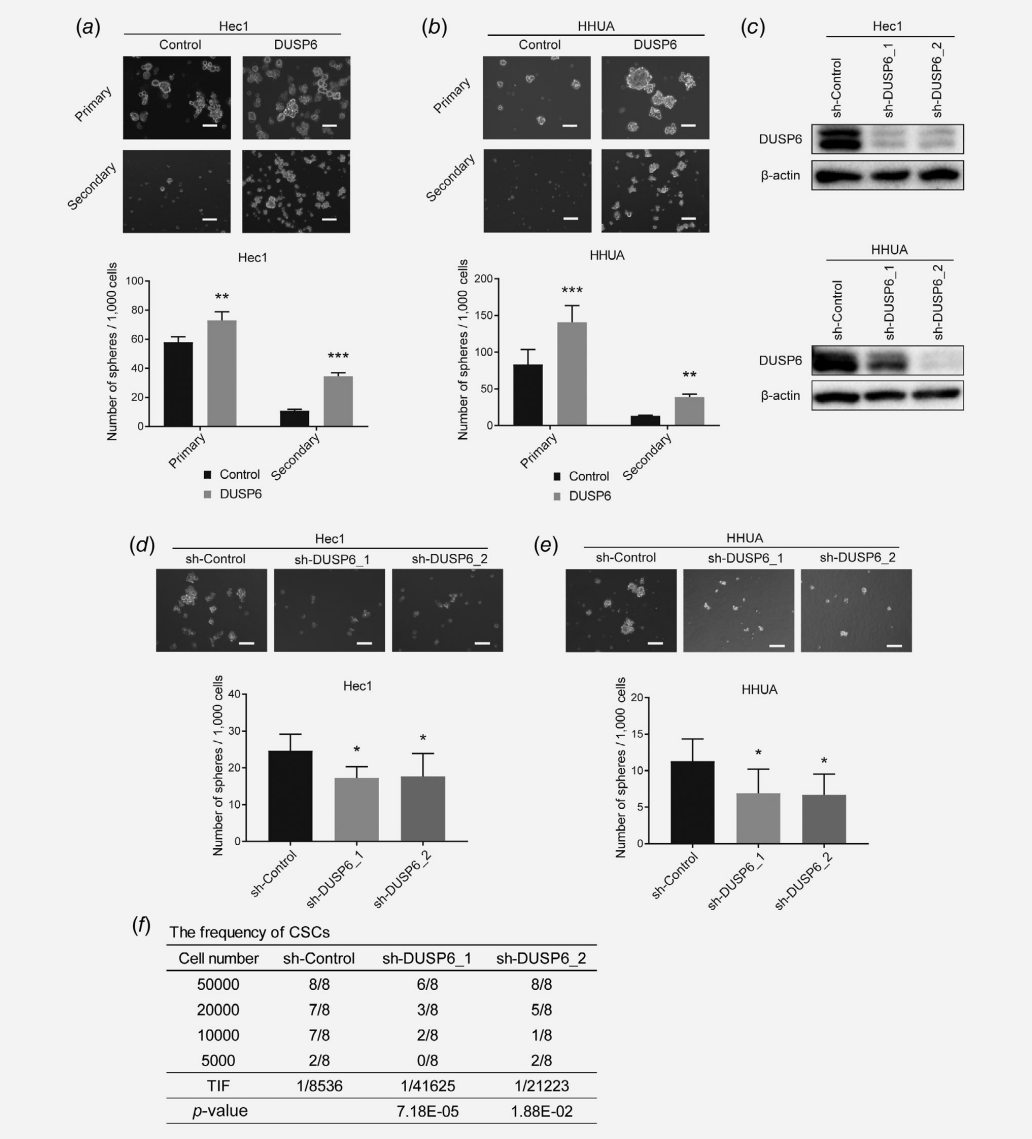
DUSP6 promotes sphere formation and self‐renewal in endometrial cancer cells. (*a*) Bright‐phase images of primary and secondary spheres formed by DUSP6‐overexpressing Hec1 cells. Scale bar, 100 μm. The bar graph shows the number of spheres/1,000 cells that were ≥50 μm in diameter. (*b*) Bright‐phase images of primary and secondary spheres formed by DUSP6‐overexpressing HHUA cells. Scale bar, 100 μm. The bar graph shows the number of spheres/1,000 cells that were ≥50 μm. (*c*) Human DUSP6 was knocked down by shRNA (sh‐DUSP6) in Hec1 and HHUA cells. The protein level of DUSP6 was measured by Western blot analysis. β‐Actin was also measured as a loading control. (*d*) Bright‐phase images of spheres formed by DUSP6‐knockdown Hec1 cells. Scale bar, 100 μm. The bar graph shows the number of spheres/1,000 cells that were ≥50 μm. (*e*) Bright‐phase images of spheres formed by DUSP6‐knockdown HHUA cells. Scale bar, 100 μm. The bar graph shows the number of spheres/1,000 cells that were ≥50 μm. (*f*) *In vivo* limiting dilution assay showing the TIF of DUSP6‐knockdown Hec1 cells. The TIF was calculated using ELDA software. Data are representative of at least three independent experiments. Error bars indicate the standard deviation. *p < 0.05, **p < 0.01, ***p < 0.005.

To investigate whether DUSP6 influences self‐renewal ability, we performed sphere formation assays in serum‐free medium. DUSP6‐overexpressing Hec1 and HHUA cells formed more spheres compared to control cells. Sequential analysis using single cells derived from the primary spheres also showed DUSP6 induced sphere formation ability in these cells. (Figs. 2 *a* and 2 *b*). Moreover, human DUSP6 expression was knocked down in Hec1 and HHUA cells using lentiviral sh‐DUSP6 (Fig. 2 *c*). Compared to cells transduced using lentiviral a nonsilencing control shRNA (sh‐Control), the DUSP6‐knockdown cells formed fewer spheres from the same number of single cells (Figs. 2 *d* and 2*e*). A role of DUSP6 in self‐renewal was confirmed by rescuing the expression of DUSP6 in siRNA‐mediated DUSP6‐knockdown cells (Supporting Information Materials and Methods). Cells were transfected with an siRNA targeting DUSP6 in the absence or presence of a DUSP6 expression plasmid. SiRNA‐mediated knockdown of DUSP6 was confirmed by Western blot analysis (Supporting Information Figs. S1 *a* and S1 *b*). The number of spheres that formed from siRNA‐mediated DUSP6‐knockdown cells treated with the DUSP6 expression plasmid was increased significantly compared to siRNA‐mediated DUSP6‐knockdown cells treated with the empty expression plasmid (Supporting Information Figs. S1 *c* and S1 *d*).

Next, we validated our results by performing a limiting dilution assay *in vivo*. We injected shRNA‐mediated DUSP6‐knockdown Hec1 cells at different inoculation densities into 6‐week‐old female NOD/SCID mice and observed tumor formation 8 weeks later. The TIF was significantly reduced in DUSP6‐knockdown cells compared to sh‐Control cells (Fig. 2 *f*). These results indicate that DUSP6 plays regulatory roles in sphere formation and self‐renewal ability in endometrial cancer.

### DUSP6 is required for invasion and metastasis of cancer cells

**Figure 3:**
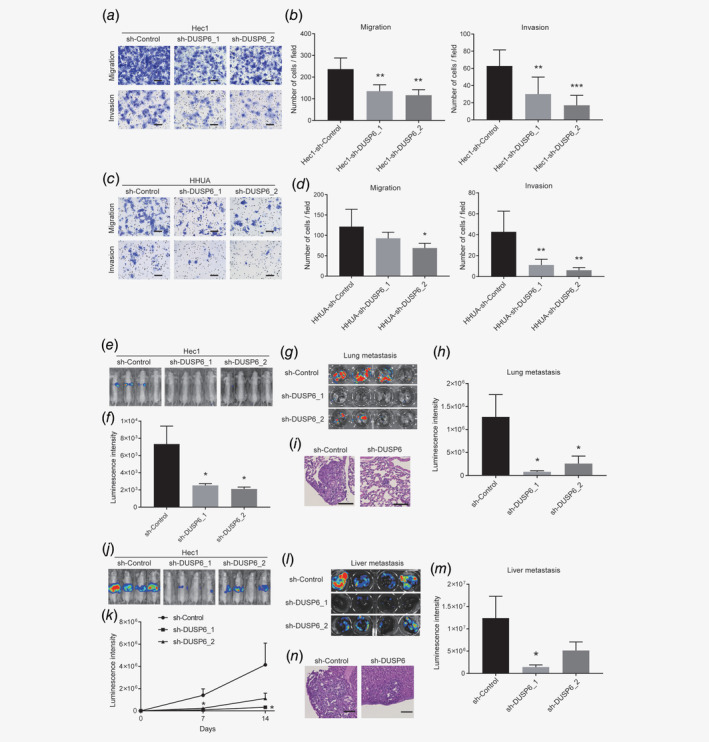
DUSP6 is required for maintaining an invasive phenotype and for promoting metastasis. (*a*) Representative bright‐phase images of migration and invasion in DUSP6‐knockdown Hec1 cells after 36 hr. Scale bar, 100 μm. (*b*) Bar graph showing quantification of the number of migrating (left) and invading (right) DUSP6‐knockdown Hec1 cells after 36 hr. (*c*) Representative bright‐phase images of migration and invasion in DUSP6‐knockdown HHUA cells after 48 hr. Scale bar, 100 μm. (*d*) Bar graph showing quantification of the number of migrating (left) and invading (right) DUSP6‐knockdown HHUA cells after 48 hr. Error bars indicate the standard deviation. Data are representative of at least three independent experiments. (*e*) Tumor volumes, assessed by the IVIS imaging system, in mice at 35 days after tail vein injection of control and DUSP6‐knockdown Hec1 cells. (*f*) Tumor volumes, quantitated by measuring luciferase activity (bioluminescence intensity), in mice at 35 days after tail vein injection. (*g*) IVIS images showing luciferase activity in the tumors of excised lungs. (*h*) Volumes of lung metastases quantitated by measuring luciferase activity (bioluminescence intensity). The volumes of tumors derived from injection of DUSP6‐knockdown cells were smaller than those derived from injection of control cells. (*i*) Representative hematoxylin‐ and eosin‐stained images of excised lungs. Scale bar, 100 μm. (*j*) Tumor volumes, assessed by the IVIS imaging system, in mice injected with intrasplenic control and DUSP6‐knockdown Hec1 cells. (*k*) Tumor volumes, quantitated by measuring luciferase activity (bioluminescence intensity), in mice after injection of intrasplenic control and DUSP6‐knockdown Hec1 cells. (*l*) IVIS image showing luciferase activity in a liver metastatic tumor. (*m*) The bar graph shows quantification of the luciferase activity in the metastatic tumor. (*n*) Representative hematoxylin‐ and eosin‐stained images of excised livers. Scale bar, 100 μm. Error bars indicate the standard error of the mean. *p < 0.05, **p < 0.01, ***p < 0.005.

The above results suggest that DUSP6 expression induces a CSC phenotype. CSCs are also responsible for cancer progression and metastasis, and we examined whether DUSP6 promotes these activities. We performed a cell invasion and migration assay using a Transwell chamber with or without extracellular matrix. ShRNA‐mediated knockdown of DUSP6 significantly reduced the abilities of Hec1 and HHUA cells to migrate in a Transwell chamber and invade the extracellular matrix layer (Figs. 3 *a*–3 *d*). We also confirmed the relationship between DUSP6 expression and invasion ability by restoring the level of DUSP6 in siRNA‐mediated DUSP6‐knockdown cells. Restoration of DUSP6 expression prevented the decrease in cell invasion caused by siRNA‐mediated DUSP6‐knockdown (Supporting Information Figs. S2 *a* and S2 *b*). Our previous findings suggested that fibronectin, which is usually associated with the extracellular matrix, is an important factor in endometrial cancer SP cells and in invasion.^15^, ^30^ The protein level of fibronectin was downregulated by shRNA‐mediated DUSP6 knockdown in Hec1 and HHUA cells (Supporting Information Fig. S3 *a*). We investigated other EMT‐related genes: N‐cadherin, vimentin and E‐cadherin. DUSP6 expression upregulated the expression of N‐cadherin, a mesenchymal cell marker, in Hec1 cells (N‐cadherin was not detected in HHUA cells). Neither vimentin nor E‐cadherin expression was influenced by DUSP6 expression (Supporting Information Fig. S3 *a* and S3 *b*).

We also assessed the requirement for DUSP6 in endometrial cancer metastasis *in vivo*. To this end, Hec1 cells stably expressing luciferase were injected into the tail vein of BALB/c‐*nu/nu* nude mice to determine whether the cells can survive and form metastatic tumors in the lung. The tumor volumes in the mice were evaluated by measuring luciferase activity, which was significantly lower in mice injected with DUSP6‐knockdown cells compared to sh‐Control cells (Figs. 3 *e* and 3*f*). In addition, the tumor volumes in excised lungs were significantly lower in mice injected with DUSP6‐knockdown cells compared to the sh‐Control cells (Figs. 3 *g*–3 *i*). These results suggest that DUSP6 plays an important role in inducing a metastatic phenotype.

We further validated our results *in vivo* by implanting Hec1 cells stably expressing luciferase into the subcapsular region of the spleen and estimated the volumes of the resulting liver metastases (Figs. 3 *j* and 3*k*). The volumes of the liver metastases derived from DUSP6‐knockdown cells using sh‐DUSP6_2 were significantly lower than those of metastases derived from the sh‐Control cells. The volumes of metastases derived from knockdown cells using a different short hairpin sequence (sh‐DUSP6_1) were also lower than those of sh‐Control‐cell‐derived metastases, although a statistically significant difference was not demonstrated (Figs. 3 *l*–3 *n*). Taken together, these results showed that DUSP6 is required for an invasive and metastatic phenotype.

### DUSP6 promotes resistance to serum starvation, inhibits apoptosis *via* Akt phosphorylation and may contribute to acquisition of a CSC phenotype

**Figure 4:**
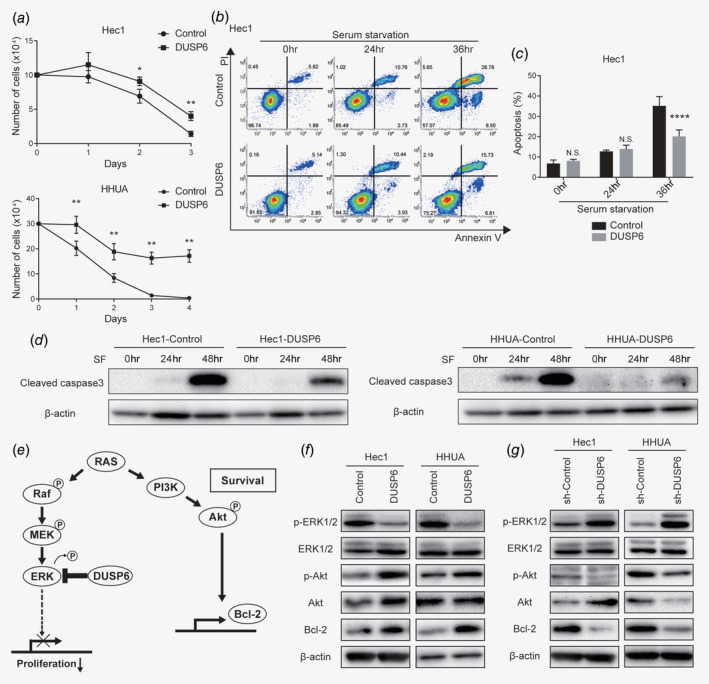
DUSP6 promotes resistance to serum starvation and inhibits apoptosis *via* Akt phosphorylation, potentially contributing to acquisition of a CSC phenotype. (*a*) Growth curves of DUSP6‐overexpressing Hec1 and HHUA cells cultured in serum‐free DMEM. Cells were counted daily. (*b*) Evaluation of apoptosis by flow cytometric analysis of annexin V in control and DUSP6‐overexpressing Hec1 cells cultured in serum‐free DMEM for the indicated number of days. The annexin V‐positive cells are gated on the right. The numbers indicate the population of each gate. (*c*) Bar graph showing the proportions of annexin V‐positive cells. (*d*) Western blot analysis of cleaved caspase 3 in control and DUSP6‐overexpressing Hec1 and HHUA cells. The cells were cultivated in serum‐free DMEM for 24 or 48 hr. β‐actin was also measured as a loading control. (*e*) Schematic of the signaling pathways that activate Akt phosphorylation and Bcl‐2, and the cross‐regulation between the Akt and MAPK signaling pathways. (*f*) Western blot analyses of p‐ERK1/2, ERK1/2, p‐Akt, Akt and Bcl‐2 in control and DUSP6‐overexpressing Hec1 and HHUA cells. β‐Actin was also measured to ensure equal loading of a gel and a representative loading control for all sample is shown. (*g*) Western blot analyses of p‐ERK1/2, ERK, p‐Akt, Akt and Bcl‐2 in sh‐Control and DUSP6‐knockdown Hec1 and HHUA cells. β‐Actin was also measured to ensure equal loading of a gel and a representative loading control for all sample is shown. Data are representative of at least three independent experiments. Error bars indicate standard deviations. *p < 0.05, **p < 0.01, ****p < 0.001, N.S., not significant.

Numerous microenvironmental factors potentially influence CSC characteristics. Nutrients may become limited in the tumor microenvironment, and nutrient depletion is an important factor among cancer cells. It has been reported that cancer cells acquire and maintain CSC characteristics in response to chronic metabolic stress.^31^ To examine whether DUSP6 is involved in acquiring resistance to serum starvation, we cultured the control and DUSP6‐overexpressing cells in serum‐free medium. Cell growth curves showed significantly better growth of DUSP6‐overexpressing cells compared to control cells (Fig. 4 *a*). To verify that the resistance to serum starvation was attributed to apoptosis inhibition, annexin V flow cytometry was performed. In control cells under the serum‐free condition, a low level of annexin V was detected at 24 hr, and this level was increased at 36 hr, whereas the annexin V level in DUSP6‐overexpressing cells was significantly suppressed (Figs. 4 *b* and 4*c*). In addition, the level of cleaved caspase 3 was also suppressed in DUSP6‐overexpressing cells at 48 hr after serum starvation (Fig. 4 *d*). These results suggest that DUSP6 inhibits apoptosis, thereby promoting resistance to serum starvation.

Our data showed that DUSP6 is involved in promoting a CSC phenotype, resistance to serum starvation and metastasis. These findings encouraged us to examine the Ras–MAPK–ERK1/2 and PI3K–Akt signaling pathways in endometrial cancer cells, especially since DUSP6 is an ERK1/2‐specific phosphatase, and both signaling pathways are activated by Ras (Fig. 4 *e*).^32^, ^33^ To assess activation of these signaling pathways, we measured phosphorylated ERK1/2 and Akt levels by performing Western blot analysis. In DUSP6‐overexpressing cells, the level of p‐ERK1/2 was decreased as reported previously,^25^ whereas the level of p‐Akt was increased (Fig. 4 *f*). In contrast, DUSP6 knockdown increased p‐ERK1/2 and decreased p‐Akt levels (Fig. 4 *g*). We also examined the level of Bcl‐2, which inhibits mitochondrial apoptosis, because Bcl‐2 expression is reportedly regulated by Akt signaling,^34^ and inhibition of apoptosis is considered a CSC phenotype. Indeed, the protein level of Bcl‐2 was upregulated in DUSP6‐overexpressing cells (Fig. 4 *f*) and downregulated in DUSP6‐knockdown cells (Fig. 4 *g*). Bcl‐2 induction appears to mediate the stress resistance induced by DUSP6 overexpression. DUSP6 induces ERK1/2 dephosphorylation and Akt phosphorylation and may contribute to acquisition of a CSC phenotype.

### High expression of DUSP6 may be associated with poor prognosis in endometrial cancers

**Figure 5:**
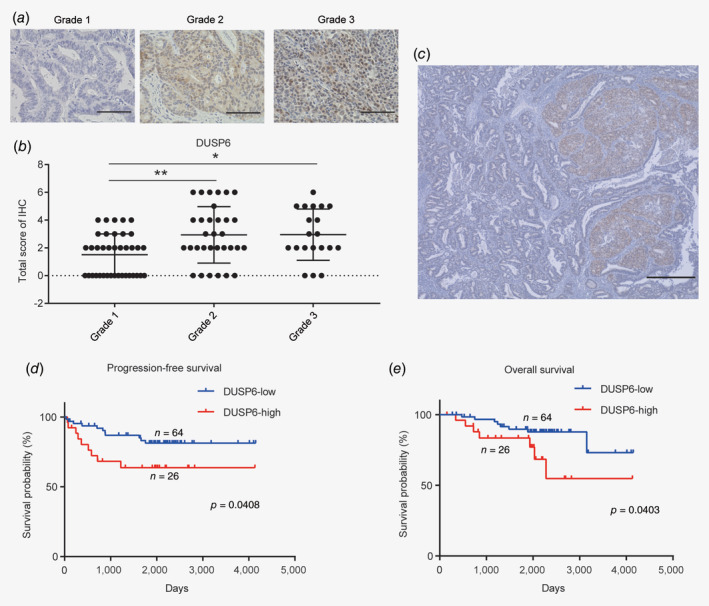
High expression of DUSP6 is potentially associated with a poor prognosis in endometrial cancer. (*a*) Representative images of IHC staining of DUSP6. Scale bar, 100 μm. (*b*) Dot plot showing the IHC scores for DUSP6 expression in Grade 1, 2 and 3 tumors. The score was significantly higher for Grade 2 and 3 than for Grade 1 tumors. (*c*) High DUSP6 expression observed in the solid portion of the tumor. Scale bar, 500 μm. (*d*) Stratification of the cases into low‐ (*n* = 64; blue line) and high‐ (*n* = 26; red line) DUSP6‐expression groups according to the DUSP6 IHC score. PFS curves were plotted using the Kaplan–Meier method and compared using the log‐rank test. (*e*) OS curves were plotted using the Kaplan–Meier method and compared using the log‐rank test. Error bars indicate the standard deviation. *p < 0.05, **p < 0.01.

To determine the clinical relevance of DUSP6, we investigated DUSP6 protein expression in 90 archived human endometrial cancer specimens using IHC. The DUSP6 IHC signals were scored according to the population of positive cells and intensity, and the total IHC score was used to evaluate the cases. Representative images of tissues from Grade 1 to 3 endometrial cancer cases are shown in Figure 5 *a*. Higher expression of DUSP6 was observed in the Grade 2 and 3 cases compared to the Grade 1 cases (Fig. 5 *b*), as well as in the poorly differentiated solid tumor portion than in the well‐differentiated luminal portion (Fig. 5 *c*). Based on the IHC total score, we then divided the 90 cases into low‐DUSP6‐expression (*n* = 64) and high‐DUSP6‐expression (*n* = 26) groups and assessed the association between DUSP6 protein expression and clinicopathological factors. High expression of DUSP6 was positively associated with the grade 2/3 tumors (*p* = 0.0052), lymph node and/or distant metastasis (*p* = 0.0270), and an advanced tumor/node/metastasis stage (*p* = 0.0309; Table 1).

**Table 1:** Clinicopathological characteristics of the patients

Characteristics	*n* (%)	DUSP6 expression		*p*‐value
		Low (*n* = 64)	High (*n* = 26)	
Patient age, years				
<50	16 (17.8%)	12 (18.8%)	4 (15.4%)	1.0000
≥50	74 (82.2%)	52 (81.2%)	22 (84.6%)	
Tumor grade				
1	38 (42.2%)	33 (51.6%)	5 (19.2%)	0.0052
2/3	52 (57.8%)	31 (48.4%)	21 (80.8%)	
Tumor invasion				
T1/T2	69 (76.7%)	52 (81.3%)	17 (65.4%)	0.1674
T3/T4	21 (23.3%)	12 (18.7%)	9 (34.6%)	
Metastasis (N1 or M1)				
No	61 (67.8%)	48 (75.0%)	13 (50.0%)	0.0270
Yes	29 (32.2%)	16 (25.0%)	13 (50.0%)	
TNM stage				
I/II	55 (61.1%)	44 (68.8%)	11 (42.3%)	0.0309
III/IV	35 (38.9%)	20 (31.2%)	15 (57.6%)	

Moreover, PFS and OS curves were plotted using the Kaplan–Meier method for the low‐ and high‐DUSP6‐expression groups. Both PFS and OS were significantly shorter in the high‐DUSP6 than in the low‐DUSP6‐expression groups (Figs. 5 *d* and 5*e*, *p* = 0.0408 and *p* = 0.0403, respectively). We also performed a subgroup analysis of PFS and OS in patients with metastasis treated with adjuvant chemotherapy. Again, PFS and OS were significantly shorter in the high‐ than low‐DUSP6‐expression groups among these patients (Supporting Information Figs. S4 *a* and S4b, *p* = 0.0205 and *p* = 0.0460, respectively). In addition, we compared DUSP6 protein levels between the primary and metastatic tumors within the same patient (*n* = 19; Supporting Information Fig. S4 *c*). It showed that DUSP6 protein levels of metastatic tumors were positively correlated with those of primary tumors (*R* = 0.8666, *p* < 0.0001), and DUSP6 expression was maintained in the metastatic tumor (Supporting Information Fig. S4 *d*; linear regression equation: *y* = 0.9359*x* − 0.5681). These results indicate that higher expression of DUSP6 is associated with high‐grade cancer and recurrence.

## Discussion

CSCs are considered to be responsible for cancer growth and relapse, and completely curing endometrial cancers requires the eradication of CSCs. Many studies have tried to identify the mechanisms contributing to CSC maintenance, including in endometrial cancers. Although DUSP6 is a negative‐feedback regulator of MAPK–ERK1/2 signaling *via* dephosphorylation, the role of DUSP6 in endometrial CSCs has not been investigated. In our study, we focused on DUSP6 functions in endometrial cancers and demonstrated that DUSP6 plays various roles related to CSC phenotype. Although abnormal activation of MAPK signaling is an important factor in cancer progression, DUSP6 influenced stemness by upregulating ALDH1, Nanog, SOX2 and Oct4A and promoted self‐renewal. DUSP6 was also positively related to apoptosis inhibition, starvation resistance, invasion, metastasis and short PFS and OS in patients with endometrial cancers.

Growing evidence has demonstrated that the PI3K–Akt pathway is a central mechanism controlling CSC features in various cancers including endometrial cancers and is strongly related to a malignant phenotype.^35^, ^36^, ^37^, ^38^ We previously demonstrated that inhibition of the MAPK pathway rescued endometrial cells from apoptosis, whereas PI3K inhibition promoted apoptosis in immortalized rat endometrial cells expressing activated KRAS proteins.^39^ Our present findings suggest that DUSP6 expression leads to inactivation of MAPK–ERK1/2 signaling and activation of PI3K–Akt. Inhibition of ERK1/2 signaling suppresses proliferation and increases the population of quiescent cells, and activation of Akt signaling promotes CSC features such as CSC‐related gene expression, self‐renewal ability, a malignant phenotype and apoptosis inhibition. Considering the demonstrated cross‐regulation between the PI3K–Akt and MAPK–ERK1/2 signaling pathways^40^, ^41^, ^42^ and identification of the key feedback loops involved in this cross‐talk,^43^ DUSP6 may play a key role in this regulation and in maintenance of a CSC phenotype.

It has been reported that the tumor microenvironment plays a role in the development of resistance to various therapies.^44^, ^45^ We suggest that DUSP6 promotes resistance to serum starvation, and high‐DUSP6‐expressing cells may adapt to microenvironments with limited nutrients. The development of recurrence and the short PFS and OS in the high‐DUSP6‐expression group among patients with metastasis who were treated with adjuvant chemotherapy may be attributed to the tumor microenvironment. We intend to analyze this possibility in future research.

Our study has some limitations. Many pathways other than PI3K–Akt signaling regulate CSC phenotypes. DUSP6 expression influenced cell invasion and the expression of EMT‐related genes, such as fibronectin and N‐cadherin, but did not influence the expression of vimentin or E‐cadherin (Supporting Information Figs. S3 *a* and S3 *b*). Recent studies indicated that cells undergo intermediate states during the transition from epithelial to mesenchymal cells. EMT occurs gradually through several cellular states expressing different levels of epithelial and mesenchymal markers and exhibiting features intermediate to those of epithelial and mesenchymal cells.^46^, ^47^, ^48^ Although EMT may require many factors other than DUSP6, DUSP6 was required for fibronectin and N‐cadherin expression and for partially inducing EMT in endometrial cancer cells.

In contrast to our findings, Fan *et al*. reported that DUSP6 inhibits EMT in the endometrial cancer cell line Ishikawa, by inhibiting migration and invasion.^49^ In their study, the viability of DUSP6‐knockdown cells was increased significantly compared to control cells, whereas in our study, the proliferation of DUSP6‐knockdown cells was slightly decreased (Supporting Information Fig. S5). However, they also showed that knockdown of DUSP6 upregulated p‐ERK, supporting our results. This discrepancy between our cell lines and Ishikawa cells might be attributed to differences in genetic backgrounds, in that Hec1 and HHUA cells possess a KRAS mutation (G12D and G12V, respectively) that Ishikawa cells lack.^50^ ERK1/2 signaling might be activated more strongly in Hec1 and HHUA cells than in Ishikawa cells. Unni *et al*. showed that in tumors with mutant oncogenes involved in the RAS pathway, ERK1/2 signaling activity is weakened to prevent toxicity and upregulation of DUSP6 may exert a protective effect on these mutant cells.^51^ DUSP6 potentially plays a more important role in RAS‐activated cell lines, such as Hec1 and HHUA cells. We intend to investigate the effect of DUSP6 in clinical cases with different genetic backgrounds.

In conclusion, DUSP6 contributed to a CSC phenotype by increasing self‐renewal ability and starvation resistance, and its expression was required for maintaining the invasive and metastatic abilities of endometrial cancer cells. In addition, in patients with endometrial cancers, DUSP6 expression may be related to short PFS and OS. These results suggest that DUSP6 has potential value as a biomarker of CSCs and as a target of therapeutics designed to eliminate CSCs in endometrial cancer.

## Conflict of interest

We have no conflicts of interest to declare.

## Supporting information


**Appendix S1**. Supporting Information.
