# Supplementary material for: Dual‐specificity phosphatase 6 plays a critical role in the maintenance of a cancer stem‐like cell phenotype in human endometrial cancer
Source: Int J Cancer. 2020 Mar 26;147(7):1987–99. doi: 10.1002/ijc.32965 (PMC7496376; doi:10.1002/ijc.32965)
Supplement: Supplementary file 1 — Appendix S1. Supporting Information. [file IJC-147-1987-s001.pdf]

**Supplementary Information**

# Dual specificity phosphatase 6 plays a critical role in the maintenance of a cancer stem-like cell phenotype in human endometrial cancer

Masaya Kato, Ichiro Onoyama, Sachiko Yoshida, Lin Cui, Keiko Kawamura, Keisuke Kodama, Emiko Hori, Yumiko Matsumura, Hiroshi Yagi, Kazuo Asanoma, Hideaki Yahata, Atsuo Itakura, Satoru Takeda, Kiyoko Kato

**Table of contents**

1. Supplementary Material and Methods
2. Supplementary Tables
3. Supplementary Figures

**Supplementary Materials & Methods**

**Transfection of an expression plasmid and siRNAs**

The coding region of the human DUSP6 gene was amplified by PCR, verified by DNA sequencing, and then inserted into the pcDNA3.1 expression vector (Addgene). The empty vector was used as a control. An siRNA was designed to target the 3' untranslated region of DUSP6 mRNA (GCCUUACCUUUGUAAAUAUtt). Silencer select negative control #1 (Ambion) was used as the control siRNA. Cells were seeded in six-well plates at 60–80% confluence and then transfected with siRNAs using Lipofectamine RNAiMAX Reagent (Thermo Fischer Scientific). After 12 hours incubation, the cells were transfected with the DUSP6 expression vector or the empty vector using Lipofectamine 2000 Reagent (Thermo Fischer Scientific). The cells were examined 24 hours after the second transfection.

**Supplementary Tables**

**Supplementary Table S1.**

Antibodies used for western blot (WB) and immunohistochemistry (IHC) analyses.

| Antigen | Source | Catalog No. | Dilution, Application |
|---|---|---|---|
| DUSP6 | Abcam Biochemicals | ab76310 | 1/1000 WB, 1/50 IHC |
| p-ERK1/2 (Thr202/204) | Cell Signaling Technology | #4370 | 1/1000 WB |
| ERK1/2 | Cell Signaling Technology | #4695 | 1/1000 WB |
| p-Akt(Ser473) | Cell Signaling Technology | #9271 | 1/1000 WB |
| Akt | Cell Signaling Technology | #9272 | 1/1000 WB |
| ALDH1/2 | Santa Cruz Biotechnology | sc-166362 | 1/100 WB |
| Bcl-2 | Cell Signaling Technology | #4223 | 1/1000 WB |
| β-actin | Santa Cruz Biotechnology | sc-81178 | 1/1000 WB |
| Cleaved caspase-3 | Cell Signaling Technology | #9661 | 1/1000 WB |
| Nanog | Cell Signaling Technology | #4903 | 1/1000 WB |
| SOX2 | Cell Signaling Technology | #3579 | 1/1000 WB |
| Oct4A | Cell Signaling Technology | #2891 | 1/1000 WB |
| Fibronectin | Abcam Biochemicals | ab23750 | 1/1000 WB |
| E-cadherin | Santa Cruz Biotechnology | sc-71009 | 1/200 WB |
| N-cadherin | Santa Cruz Biotechnology | sc-7939 | 1/200 WB |
| Vimentin | Santa Cruz Biotechnology | sc-6260 | 1/200 WB |

**Supplementary Table S2.** Scoring system for immunohistochemical staining.

| Score for proportion of positive cells | | Score for staining intensity | | Total score |
|---|---|---|---|---|
| 0 | 0% | 0 | negative | Sum of the two scores |
| 1 | 1–33% | 1 | low | |
| 2 | 34–66% | 2 | moderate | |
| 3 | 67–100% | 3 | high | |

**Supplementary Figures**

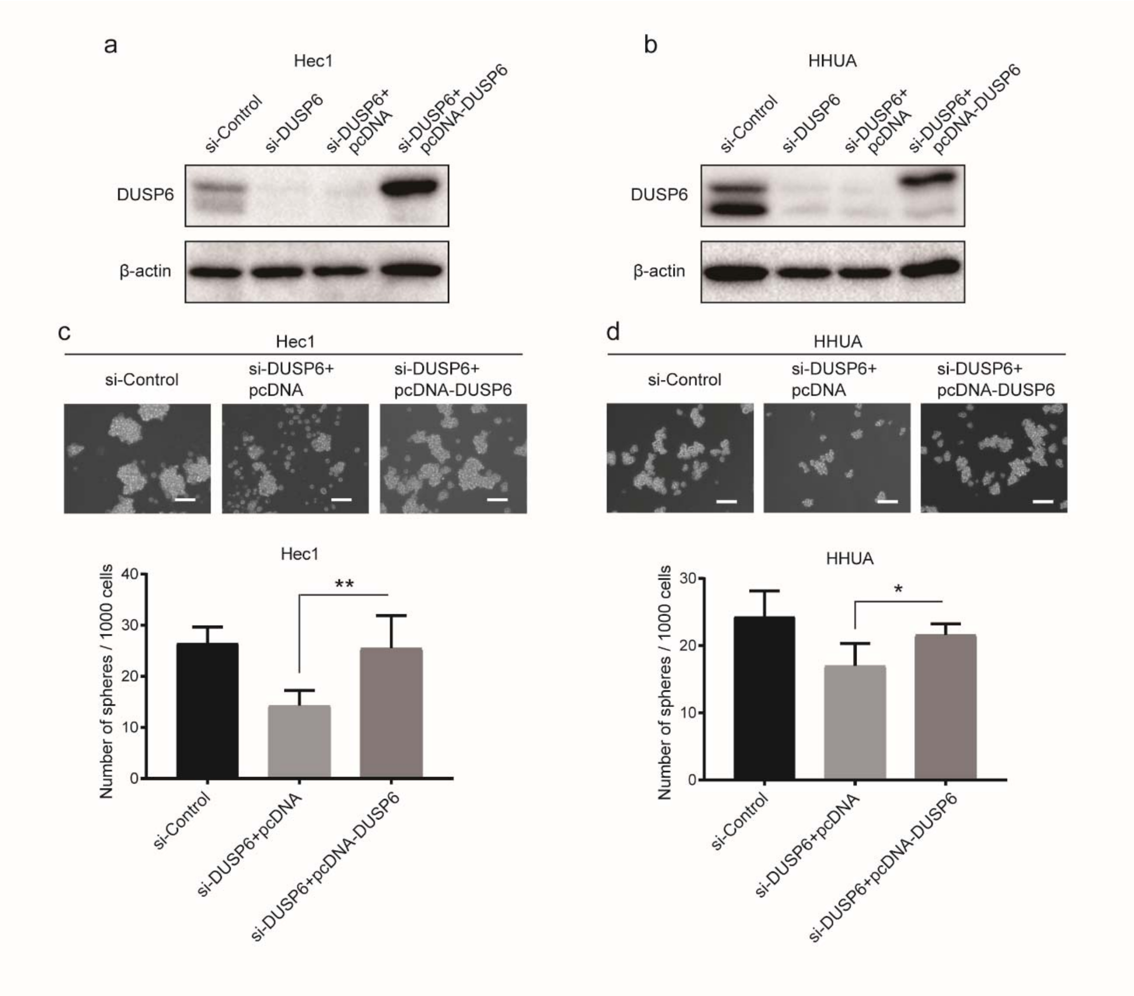


**Supplementary Figure S1.**

**a, b** Hec1 and HHUA cells transfected with the siRNA targeting DUSP6 in the absence or presence of the DUSP6 expression vector. The protein level of DUSP6 was measured by Western blot analysis. β-actin was also measured as a loading control. **c** Bright-phase images of spheres formed by Hec1 cells transfected with the DUSP6 siRNA in the absence or presence of the DUSP6 expression vector. Scale bar, 100 μm. The bar graph shows the number of spheres/1000 cells that were ≥50 μm in diameter. **d** Bright-phase images of spheres formed from HHUA cells transfected with the DUSP6 siRNA in the absence or presence of the DUSP6 expression vector. Scale bar, 100 μm. The bar graph shows the number of spheres/1000 cells that were ≥50 μm in diameter. Data are representative of at least three independent experiments. Error bars indicate the standard deviation. *P<0.05, **P<0.01.

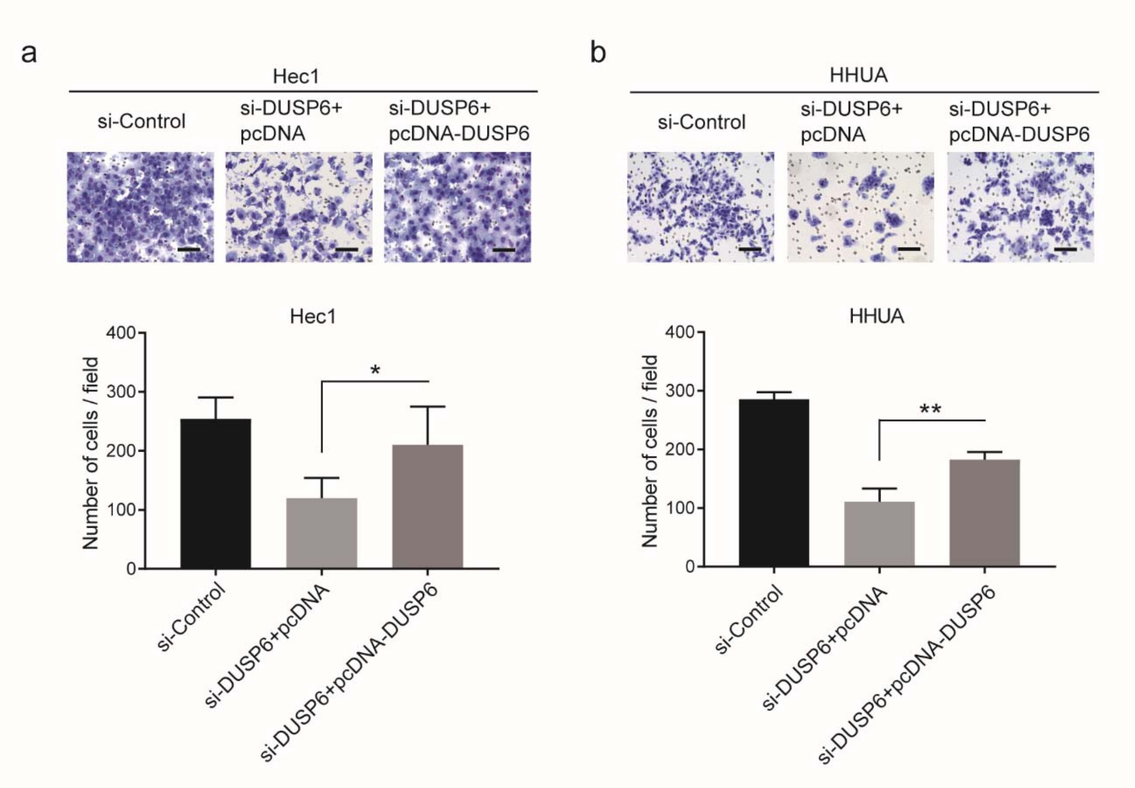


**Supplementary Figure S2.**

**a** Representative bright-phase images of invading Hec1 cells transfected with the DUSP6 siRNA in the absence or presence of the DUSP6 expression vector after 36 hours. Scale bar, 100 μm. Bar graph of the number of invading cells after 36 hours. **b** Representative bright-phase images of invading HHUA cells transfected with the DUSP6 siRNA in the absence or presence of the DUSP6 expression vector after 48 hours. Scale bar, 100 μm. Bar graph of the number of invading cells after 48 hours. Data are representative of at least three independent experiments. Error bars indicate the standard deviation. *P<0.05, **P<0.01.

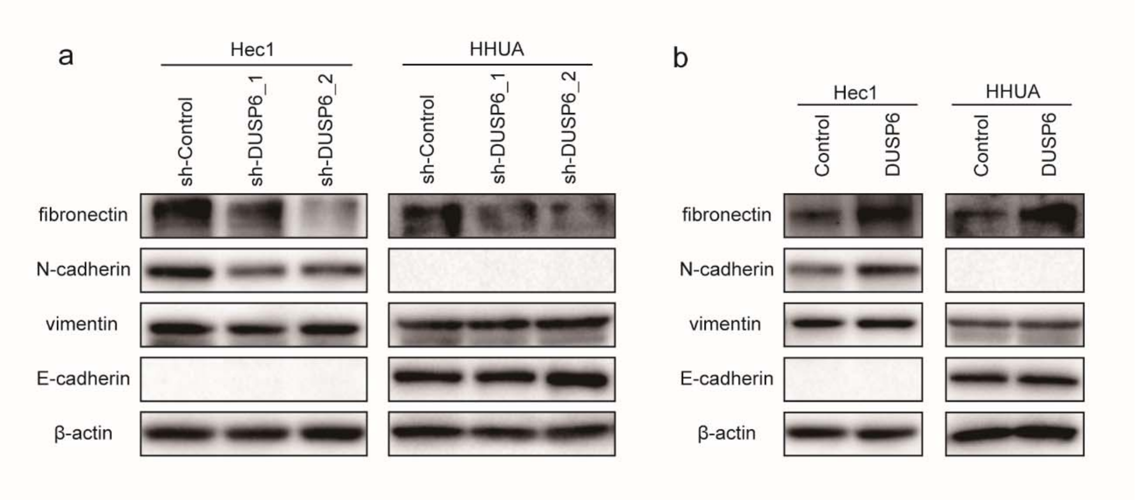


**Supplementary Figure S3.**

**a** Western blot analyses of fibronectin, N-cadherin, vimentin and E-cadherin in sh-Control- and sh-DUSP6-transducted Hec1 and HHUA cells. β-actin was also measured to ensure equal loading of a gel and a representative loading control for all sample is shown. **b** Western blot analyses of fibronectin, N-cadherin, vimentin and E-cadherin in control and DUSP6-overexpressing Hec1 and HHUA cells. β-actin was also measured to ensure equal loading of a gel and a representative loading control for all sample is shown. Data are representative of at least three independent experiments.

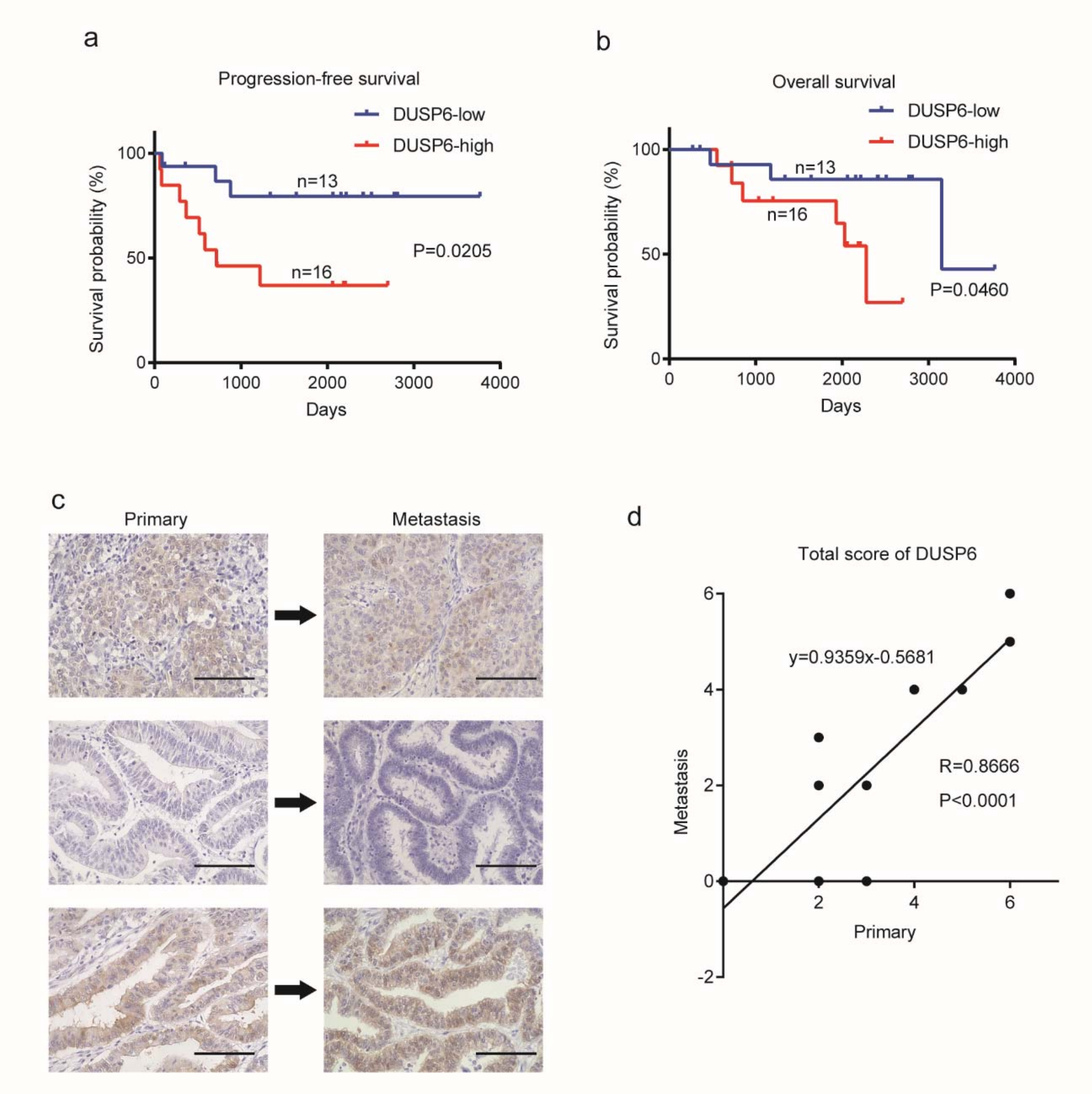


**Supplementary Figure S4.**

**a** Cases with lymph node or distant metastases treated with chemotherapy after surgery, stratified into low- (n=13; blue line) and high- (n=16; red line) DUSP6-expression groups according to the DUSP6 IHC score. PFS curves were plotted using the Kaplan–Meier method and compared using the log rank test. **b** OS curves were plotted using the Kaplan–Meier method and compared using the log rank test. **c** Representative images of IHC staining of DUSP6, showing the primary and metastatic lesions within the same patients. Scale bar, 100 µm. **d** Dot plot and linear regression analysis of DUSP6 expression between the primary and metastatic lesions of the same patient (n=19). The x-axis and y-axis show the DUSP6 expression level in the primary and metastatic lesions, respectively. Linear regression equation: $y = 0.9359x - 0.5681$.

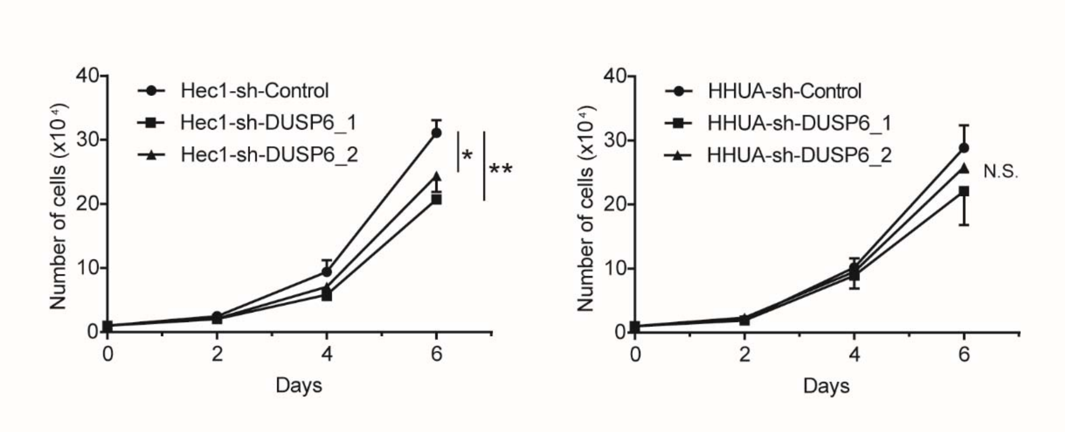


**Supplementary Figure S5.**

Growth curves of DUSP6-knockdown Hec1 and HHUA cells. A total of $1.0 \times 10^4$ cells were plated in DMEM containing 10% FBS, and the number of cells was counted every other day. Data are representative of at least three independent experiments. Error bars indicate the standard deviation. *P<0.05, **P<0.01; N.S., not significant.
